# The short term impact of radiofrequency ablative techniques and peripheral nerve block on thermoregulation in mouse models

**DOI:** 10.1038/s41598-024-82049-8

**Published:** 2024-12-28

**Authors:** Tomoo Yuba, Yoshihisa Koyama, Yuki Kinishi, Yuji Fujino, Shoichi Shimada

**Affiliations:** 1 Department of Anesthesiology and Intensive Care Medicine, Osaka University Graduate School of Medicine, Suita, Japan; 2 Department of Neuroscience and Cell Biology, Osaka University Graduate School of Medicine, Osaka, 565-0871 Japan; 3 Addiction Research Unit, Osaka Psychiatric Research Center, Osaka Psychiatric Medical Center, Osaka, 541-8567 Japan; 4 Global Center for Medical Engineering and Informatics, Osaka University, Suita, 565-0871 Japan; 5 Integrated Frontier Research for Medical Science Division, Institute for Open and Transdisciplinary Research Initiatives (OTRI), Osaka University, Suita, 565-0871 Japan

**Keywords:** Thermoregulation, Peripheral nerve block, Local anesthesia, Conventional radiofrequency thermocoagulation, Pulsed radiofrequency, The preoptic area, Neuroscience, Peripheral nervous system

## Abstract

This study investigated the impact of multiple nerve block methods (local anesthesia, conventional radiofrequency thermocoagulation [CRF], and pulsed radiofrequency [PRF]) on thermoregulation. Focusing on hypothalamic function, the effects of local anesthesia, CRF, and PRF on central and peripheral temperatures were analyzed and compared. Our findings revealed that all three nerve block groups cause a decrease in central temperature, with the CRF group exhibiting the most pronounced effect. Furthermore, immunostaining analysis showed decreased neural activity in the preoptic area, suggesting that nerve blocks may influence central thermoregulatory mechanisms. This study provides valuable insights into the effects of peripheral nerve blocks on thermoregulation and may contribute to the development of therapeutic strategies to managing perioperative hypothermia and enhancing pain management, especially in patients undergoing surgeries with high risks of thermoregulatory complications, such as on-pump surgery and laparoscopic surgery.

## Introduction

The regulation of body temperature is vital for optimal physiological function. Neurons in the hypothalamus serve as the thermoregulatory center, detecting changes in body temperature and responding appropriately to uphold thermal equilibrium^1^. While the effect of general anesthesia on body temperature is well recognized, epidural and spinal anesthesia methods, typically devoid of direct drug exposure to the brain, have also been implicated in impaired thermoregulation and lowering overall body temperature^2^. These methods result in the blocking afferent pain signaling and efferent nerves that control vasoconstriction and shivering, ultimately leading to reduced body temperature^3^. However, the effects of peripheral nerve blockade, wherein local anesthesia is administered at a more peripheral site compared to epidural or spinal anesthesia, on thermoregulation remain to be fully elucidated.

This study aimed to investigate the effect of different nerve-blocking techniques (local anesthesia, conventional radiofrequency thermocoagulation [CRF], and pulsed radiofrequency [PRF]) on thermoregulation. CRF involves the application of continuous electrical current to heat tissue and disrupt nerve function, typically used for chronic pain conditions where other treatments have failed. It is indicated for conditions such as facet joint pain, trigeminal neuralgia, and chronic low back pain. On the other hand, PRF is a minimally invasive method that delivers intermittent electrical pulses to nerve tissues, causing less neural damage and is indicated for conditions like neuropathic pain and radicular pain. Both techniques aim to modify the pain signals before they reach the brain, potentially influencing thermoregulatory pathways by altering peripheral nerve inputs.

## Results

**Fig. 1:**
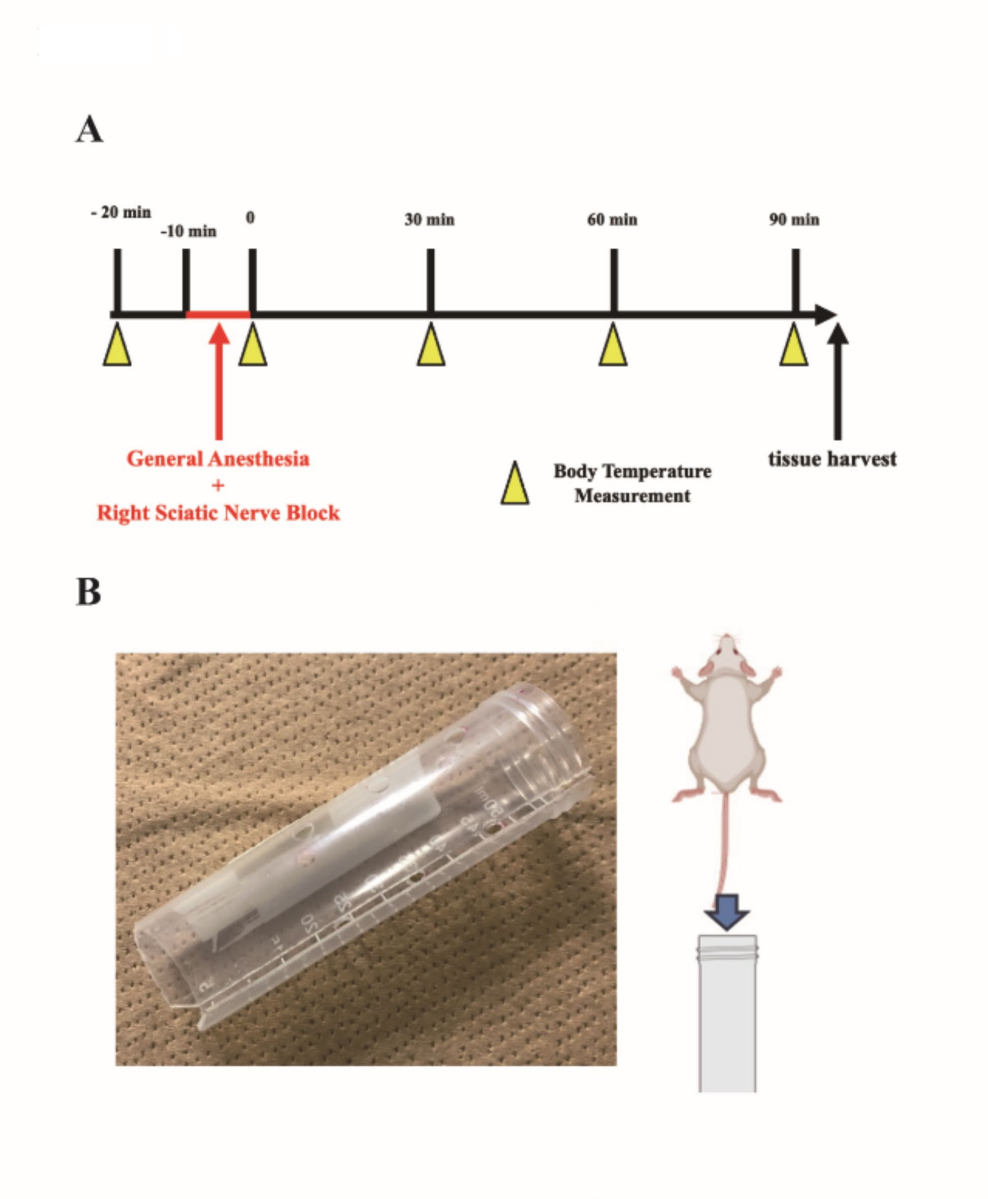
(**a**) Experimental protocol. The procedure was performed under general anesthesia with 1.5% isoflurane for 10 min. Brains were harvested at 90 min after nerve block or sham operation. (**b**) Body temperature was measured using a modified 50-ml centrifuge tube in which the mouse was restrained. A groove was made in the tube to allow the mouse tail to pass through, and the mouse was gently placed in the tube for a quick temperature measurement. Created using BioRender Com.

Given the necessity for immobilization of the mice during the procedure, nerve blocks were conducted under general anesthesia with isoflurane (Fig. 1a, b). However, it is important to note that general anesthesia with isoflurane significantly impairs thermoregulation and concurrently lowers the thresholds for vasoconstriction and shivering^2^. Therefore, a comparative analysis of the sham and nerve block groups was performed to evaluate central and peripheral temperatures, aiming to determine the timing of recovery from the temperature reduction induced by isoflurane.

### Chronological alteration of body temperature

**Fig. 2:**
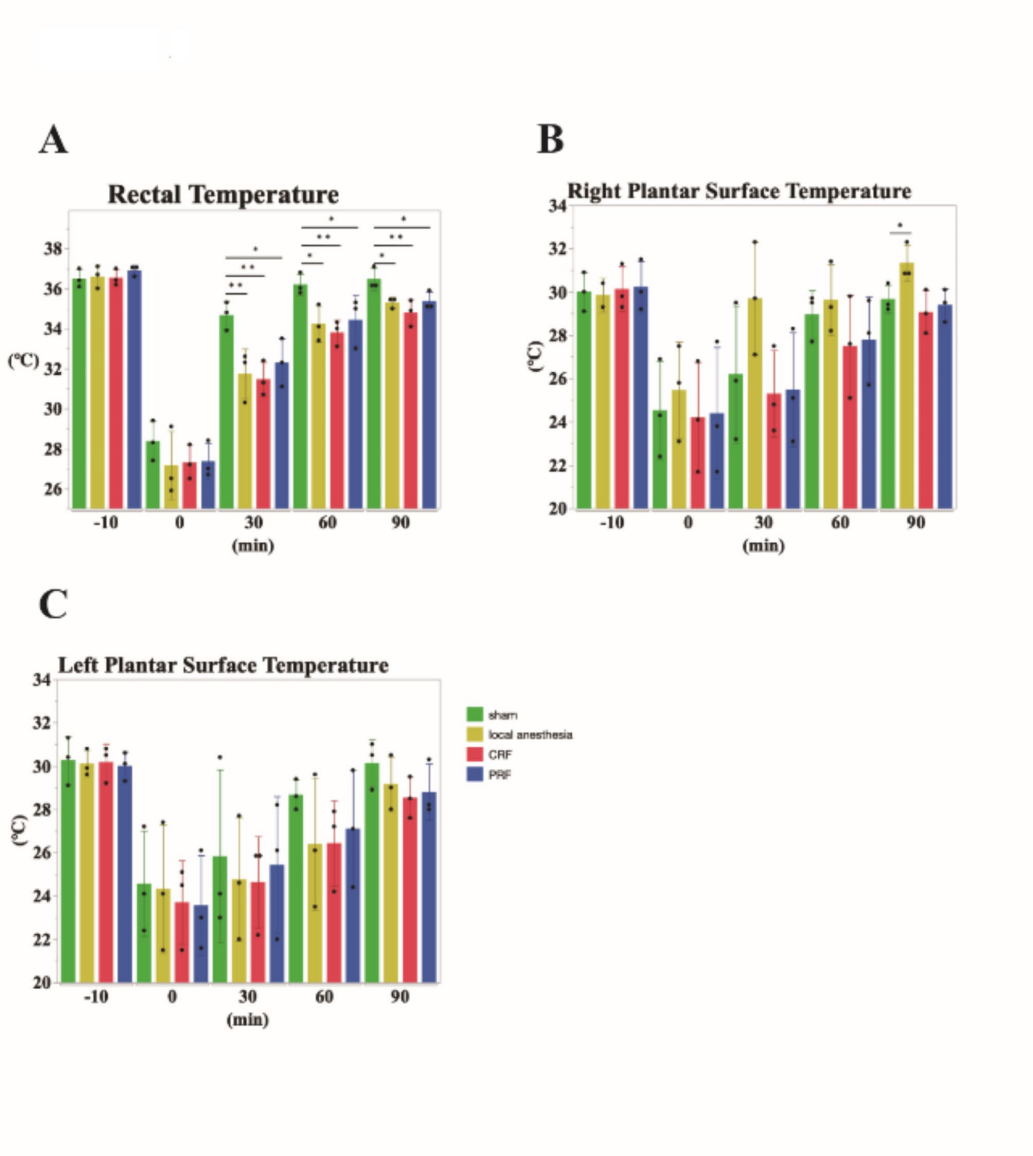
(**a**) Bar graph for average of rectal temperature. Sham group (green), local anesthesia group (yellow), CRF (red), and PRF (blue). Temperatures at -10 min were not different in each group. Compared to -10 min, body temperature at 0 min was significantly lower in all group, and then recovered over time. However, the nerve block groups recovered more slowly than the sham group at the same time points: at 30 min, the local anesthesia group (*p* = 0.0083), CRF group (*p* = 0.0052), and PRF group (*p* = 0.023); at 60 min, the local anesthesia group (*p* = 0.025), CRF group (*p* = 0.0099), and PRF group (*p* = 0.038 ), and at 90 min, the local anesthesia group (*p* = 0.022), CRF group (*p* = 0.0037), and PRF group (*p* = 0.028). (**b**, **c**) Bar graph of the average right (b) and left hind paw (**c**) planter surface temperatures. Sham (green), local anesthesia (yellow), CRF (red), and PRF (blue). Temperatures at 10 and 0 min were not different in all groups. (**b**) On the right hind paw planter surface, recovery was slower in the CRF and PRF groups than in the sham group; however, in the local anesthesia group, the temperature had already recovered to the same level as before treatment after 30 min and was higher than before treatment after 90 min (90 min local anesthesia group, *p* = 0.038). (**c**) On the left hind paw planter surface, the nerve block group showed slower temperature recovery than the sham group.

Before treatment, no significant differences were observed in central and peripheral temperature between the groups (Fig. 2a: rectal temperature: sham group, 36.5 ± 0.46 °C; local anesthesia group, 36.6 ± 0.56 °C; CRF group, 36.5 ± 0.42 °C; PRF group 36.9 ± 0.26 °C; Fig. 2b: right hind paw: sham group, 30.0 ± 0.9 °C; local anesthesia group, 29.9 ± 0.78 °C; CRF group, 30.1 ± 1 °C; PRF group, 30.2 ± 1.06c°C; Fig. 2c: left hind paw: sham group, 30.3 ± 1.11 °C; local anesthesia group, 30.1 ± 0.62 °C; CRF group, 30.2 ± 0.85 °C; PRF group, 30.0 ± 0.66 °C). However, following the administration of general anesthesia, the central and peripheral temperatures decreased significantly in all groups (Fig. 2a: rectal temperature: sham group, 28.4 ± 1.00 °C; local anesthesia group, 27.2 ± 1.70 °C; CRF group, 27.3 ± 0.85 °C; PRF group, 27.4 ± 0.91 °C; Fig. 2b: right hind paw: sham group, 24.5 ± 2.26 °C, local anesthesia group, 25.5 ± 2.22 °C, CRF group, 24.2 ± 2.55 °C, PRF group, 24.4 ± 3.04 °C; Fig. 2c: left hind paw: sham group, 24.6 ± 2.43 °C, local anesthesia group, 24.3 ± 2.96 °C, CRF group, 23.7 ± 1.93 °C, PRF group, 23.6 ± 2.30 °C).

At 60 min post-treatment, the central temperature of the sham group had recovered to pre-treatment level; however, all nerve block groups exhibited significantly lower central temperatures compared to the sham group (Fig. 2a: sham group, 36.2 ± 0.53 °C, local anesthesia group, 34.2 ± 0.91 °C, *p* = 0.025; CRF group, 33.8 ± 0.62 °C, *p* = 0.0099; PRF group, 34.4 ± 1.25 °C, *p* = 0.038). Even at 90 min post-treatment, all nerve block groups exhibited significantly lower central temperatures than the sham group (Fig. 2a: sham group, 36.5 ± 0.55 °C; local anesthesia group, 35.3 ± 0.26 °C, *p* = 0.022; CRF group, 34.8 ± 0.66 °C, *p* = 0.0037; PRF group, 35.4 ± 0.46 °C, *p* = 0.028). Among the nerve block groups, the CRF group exhibited the most pronounced decrease (Fig. 2a).

Immediately after treatment, the right hind paw surface temperature decreased in all the groups (Fig. 2b). The right peripheral temperature of the sham group gradually increased and recovered to near pre-treatment level at 60 min post-treatment. The right peripheral temperature of CRF and PRF groups also gradually increased over time and recovered to near pre-treatment level at 90 min (Fig. 2b: sham group, 29.7 ± 0.64 °C; CRF group, 29.1 ± 1.00 °C, *p* = 0.40; PRF group, 29.4 ± 0.75 °C, *p* = 0.70). In contrast, the right peripheral temperature of local anesthesia group was higher than that of sham group at the same time point, and this trend persisted until 90 min post-treatment (Fig. 2b: local anesthesia group, 31.3 ± 0.84 °C, *p* = 0.038).

The left hind paw surface temperature also decreased after treatment in all groups (Fig. 2c). The left peripheral temperature of the sham group gradually increased and recovered to near pre-treatment levels after 90 min. The left peripheral temperature of three nerve block groups also gradually increased and was slightly lower than that of the sham group after 90 min (Fig. 2c: sham group, 30.1 ± 1.10 °C, local anesthesia group, 29.2 ± 1.26 °C, *p* = 0.34; CRF group, 28.5 ± 0.95 °C, *p* = 0.13; PRF group, 28.8 ± 1.3 °C, *p* = 0.20) (Fig. 2c).

### Neural activity in the median preoptic nucleus, the thermoregulatory center

**Fig. 3:**
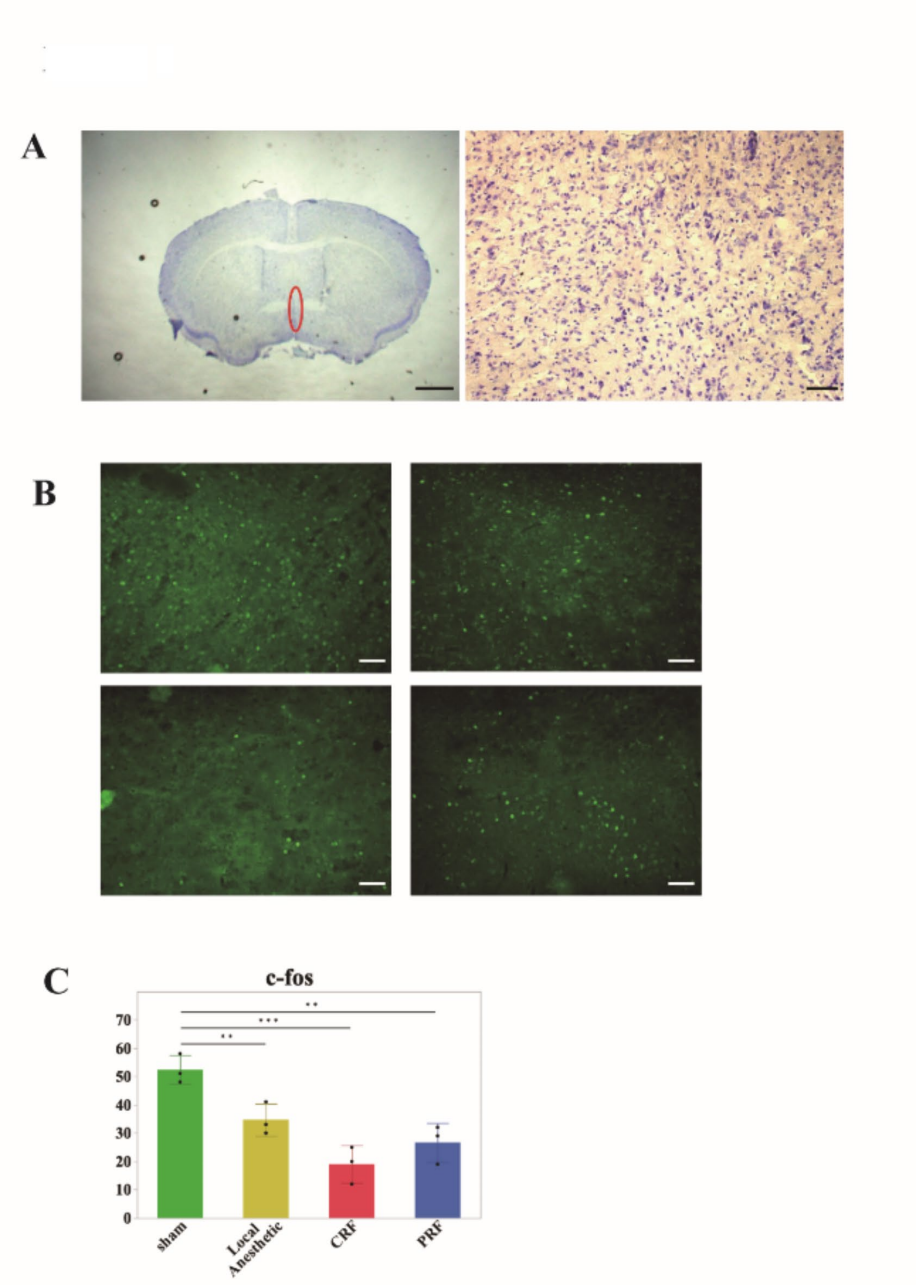
(**a**) Micrograph of Nissle-stained image. Left: low magnification, right: high magnification. Neuronal activity was evaluated in the MnPO circled in red. Scale bar: 1 mm (left), 50 μm (right). (**b**) Representative immunostaining photomicrograph of each group. Upper left: sham group, upper right: local anesthesia group, lower left: CRF group, and lower right: PRF group. Scale bar: 50 μm. (**c**) Bar graph for number of c-Fos-positive neurons. Sham group (green), local anesthesia group (yellow), CRF (red), and PRF (blue). The number of the MnPO neurons in each group were significantly lower than sham group (local anesthesia group *p* = 0074, CRF group *p* < 0.001, PRF group *p* = 0.0039).

To examine the neural activity in the MnPO (Fig. 3a), 90 min after nerve block treatment, we performed immunostaining using a c-FOS antibody, a neural activity marker. The number of c-FOS-positive cells in the MnPO was the highest in the sham group, followed by the local anesthesia and PRF groups, and lowest in the CRF group (Fig. 3b and c: 52.3 ± 5.13 in the sham group; 34.7 ± 5.69 in the local anesthesia group, *p* = 0.0074; 19.0 ± 6.55 in the CRF group, *p* < 0.001; 26.7 ± 6.81 in the PRF group, *p* = 0.0039).

### Extended Observation to 48 h

**Fig. 4:**
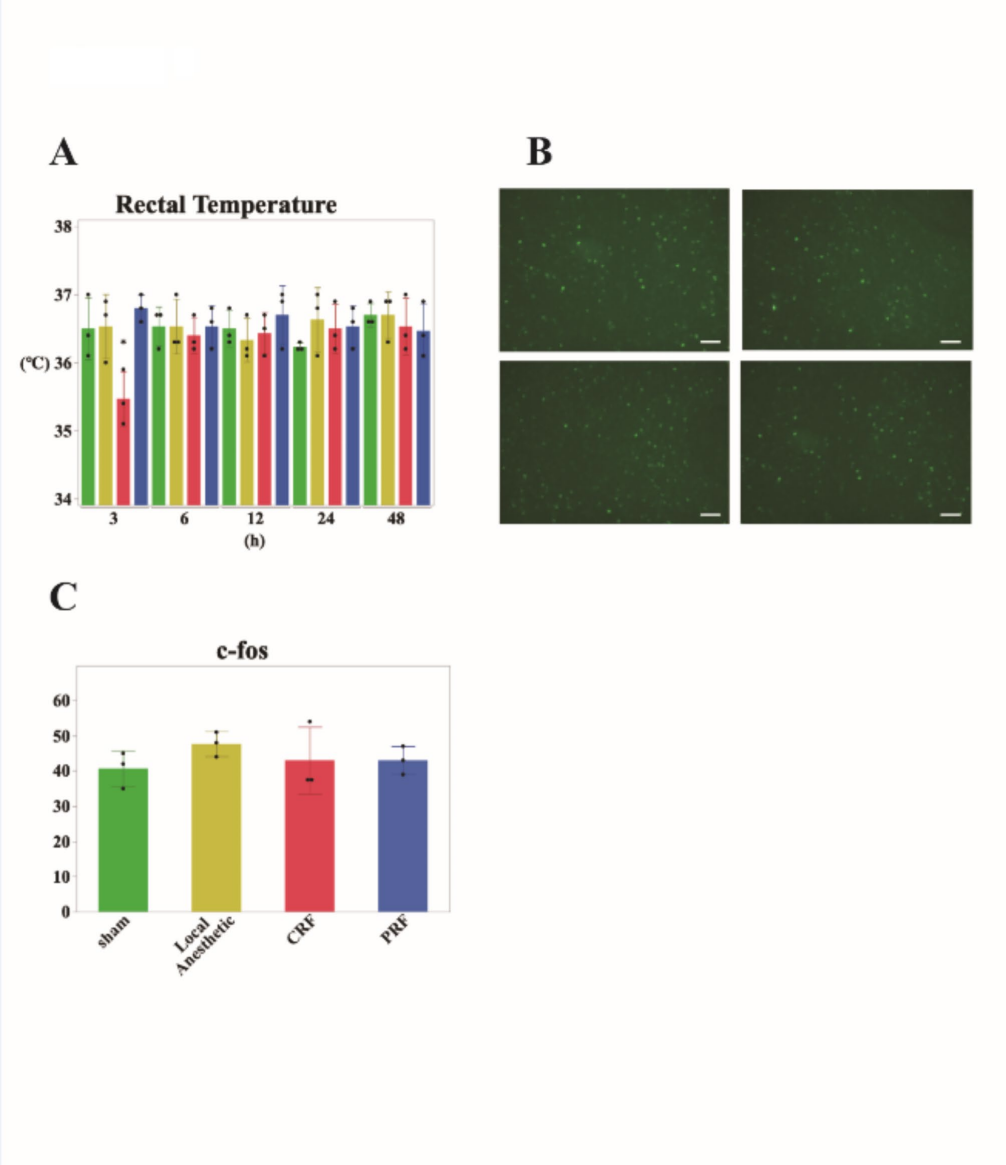
(**a**) Rectal temperatures observed over a 48-hour period. Sham group (green), local anesthesia group (yellow), CRF (red), and PRF (blue). This graph shows that while the CRF group exhibited a temporary decrease in rectal temperature at three hours (*p* = 0.044), there were no enduring differences in temperature across any groups thereafter. Additionally, no differences were observed in the plantar temperatures of the left and right hind paws in any group after 3 h. (**b**) Representative immunostaining photomicrograph of each group. Upper left: sham group, upper right: local anesthesia group, lower left: CRF group, and lower right: PRF group. Scale bar: 50 μm. (**c**) Bar graph for number of c-Fos-positive neurons. No significant differences were observed in the number of MnPO neurons among the groups.

Finally, we observed changes in body temperature in each group during the chronic phase from the initial 90 min to 48 h. The trends in rectal temperature up to 90 min were consistent with previous observations. However, only the CRF group showed a significant decrease in body temperature compared to the sham group at three hours post-treatment (*p* = 0.044), after which no differences were observed between any of the groups (Fig. 4a). The plantar temperatures of the hind paws showed no significant differences at any time point. At 48 h of post-treatment, the neural activity in the MnPO was examined. There was no significant difference in c-Fos activity in the MnPO across all groups (Fig. 4b, c).

## Discussion

In this study, we investigated the effects of different nerve block methods (local anesthesia, CRF, and PRF) on body temperature using mice. Similar to previous reports, the administration of general anesthesia resulted in decrease in body temperature^4^. Surprisingly, a decrease in the central temperature was observed in radiofrequency ablative techniques and peripheral nerve block groups. Furthermore, immunostaining with c-Fos antibody revealed that the number of active neurons in the MnPO, the thermoregulatory center, was significantly decreased in the radiofrequency ablative techniques and peripheral nerve block group. These findings suggest that nerve blockade leads to decreased central and peripheral body temperature and decreased neural activity of the MnPO may be involved in this temperature reduction.

**Fig. 5:**
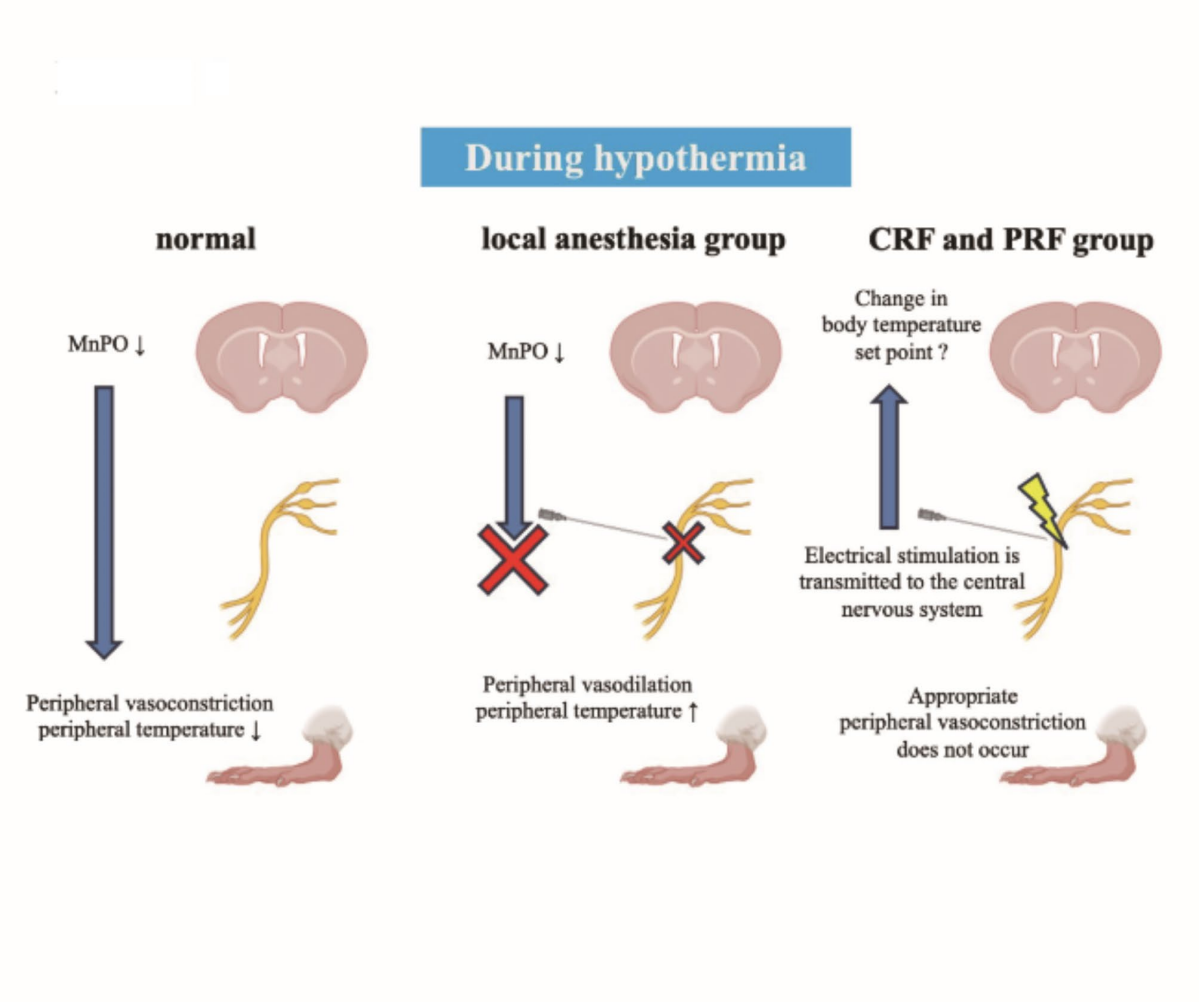
The effects of each nerve block group during hypothermia. Nerve blocks may act not only on peripheral nerves but also on central nervous system. Created using BioRender Com.

We performed three types of nerve blocks with different mechanisms of action. Local anesthesia suppresses nerve activity by inhibiting neural depolarization (Ion Channels of Excitable Membranes). CRF thermally denervates by generating continuous radiofrequency waves from the tip of the needle to generate a high temperature (80 °C) at the needle tip^5^. PRF involves the intermittent generation of radio waves to maintain the needle tip at approximately 40 °C to induce nerve block^6,7^. Although the detailed mechanism of action remains unclear, PRF is an effective method for treating neuropathic pain because it is less likely to cause hypoesthesia, unlike local anesthesia and CRF^8^. Both PRF and CRF have been reported to be effective for chronic pain involving sympathetic nerves, suggesting that they may act on the sympathetic system^9^. This implies that both PRF and CRF could influence body temperature, as evidenced by the temperature changes observed in this study when compared to the sham group. However, the specific mechanisms through which they affect thermoregulation remain unclear, necessitating further research. Our results revealed that the three nerve block methods had different effects on the central and peripheral temperatures. Notably, the right hind paw surface temperature after the procedure was higher in the local anesthesia group than in the other groups. This result is consistent with the fact that epidural and spinal anesthesia block the autonomic nervous system and inhibit vasoconstriction. Although the sciatic nerves treated in this study are primarily composed of sensory and motor fibers, they do contain some autonomic fibers as well^10^, which may explain why the hind paw surface temperature of the right foot was higher in the local anesthesia group compared to the other groups. Since local anesthetics tend to infiltrate the surrounding tissues, it is speculated that they may block a wide range of nerves, including autonomic nerves^11^. This infiltration might explain why the CRF group, which is supposed to induce permanent nerve degeneration, showed differences in peripheral temperature compared to the local anesthesia group. The autonomic nervous system is not only contained within the sciatic nerve but also extensively distributed directly from preganglionic fibers, with its pathways being quite diverse^11^. The response to hypothermia is to inhibit the release of body heat, typically by constricting the peripheral blood vessels. In the local anesthesia group, the administered local anesthetic blocked a wide range of autonomic nerves and inhibited peripheral vasoconstriction, which could potentially affect body temperature. However, a decrease in body temperature was observed after 90 min post-treatment in the CRF and PRF groups, without clear inhibition of peripheral vasoconstriction. This suggests that nerve blocks may impact central thermoregulation through a mechanism other than autonomic nerve blockade (Fig. 5). The observation of reduced body temperature following nerve block, alongside decreased activity in the MnPO, prompts consideration of alternative explanations such as sensory input disruption or vasodilation-induced heat loss. The role of peripheral nerve input in central temperature regulation is complex and involves multiple pathways and feedback mechanisms. As demonstrated by Morrison and Nakamura, the hypothalamus integrates various sensory inputs to maintain thermal homeostasis^12,13^.

The sham group recovered its central temperature at 60 minutes post-treatment, whereas the nerve block group showed no such recovery. Reports typically suggest that nerve blocks performed far from the central nervous system, as in this study, do not usually affect the overall body temperature^14^, contrasting with the results observed in our research. This difference may be attributed to the nerve block technique. The local anesthesia blocks nerve transmission by suppressing neural depolarization, whereas CRF blocks nerve transmission via thermal denervation. The sensory blockade induced by local anesthesia and CRF primarily affects the afferent nerve fibers that carry temperature and pain signals to the central nervous system. The hypothalamus integrates various sensory inputs, including temperature, to maintain homeostasis. Under normal conditions, it receives constant updates on peripheral temperature, which it uses to initiate compensatory mechanisms such as vasodilation or vasoconstriction, shivering, and sweating to maintain core temperature. When peripheral sensory inputs are diminished due to nerve blocks, the hypothalamus may not receive accurate temperature signals, leading to inappropriate thermal responses. Research has shown that thermoregulation is critically dependent on the integrity of afferent and efferent pathways within the autonomic nervous system. For instance, studies have highlighted how regional anesthesia can lead to hypothermia by blocking sympathetic nerve activity, which is crucial for vasoconstriction and shivering responses^14^. Moreover, the inactivation of sensory inputs can lead to an ‘underestimation’ of peripheral temperatures, causing a shift in the hypothalamic set point for temperature regulation^15^. Additionally, the role of neurotransmitters such as glutamate, which is significant in mediating the sensory transmission to the hypothalamus, cannot be overlooked. Disruption in glutamatergic transmission due to nerve blocks might impede the hypothalamus’s ability to accurately gauge and respond to changes in body temperature^13^. Taken together, these two groups may have had reduced sensory stimulation from the area innervated by the blocked nerve, thereby affecting central thermoregulation.

Surprisingly, the PRF group, which supposedly did not experience a decreased sensation^16^, also showed a decrease in body temperature. PRF may act centrally, without altering the activity of thermosensitive receptors. CRF and PRF have been shown to affect nerve fibers differently. CRF, by generating high temperatures, can cause extensive coagulative necrosis affecting all types of nerve fibers, including C fibers and Aδ fibers, which are primarily involved in pain transmission and temperature sensation^5^. PRF, meanwhile, by delivering electrical energy intermittently, might preserve the structure of the nerve while still modulating its function, potentially affecting the signaling pathways without complete denervation^6^. Therefore, PRF may exert mechanisms other than peripheral neuronal control, which may lead to the development of new therapeutic strategies.

MnPO is an important neuronal region involved in maintaining body temperature homeostasis, the greatest characteristic of homothermal animal, through autonomic thermoregulatory responses such as heat dissipation and production^17^. MnPO not only monitors core body temperature (brain temperature), but also receives environmental temperature information from temperature sensors in the skin via sensory nerves. In fact, when body temperature is lowered, the neurons expressing prostaglandin EP3 receptor in the MnPO are activated, suppressing heat dissipation and production by inhibiting sympathetic output. When body temperature is increased, the GABAergic inhibitory neurons in the MnPO are activated, which induces heat production and maintains thermostability through sympathetic nerve activation. Thus, when body temperature is regulated, neural activity is always observed in the MnPO. Our findings revealed that the number of activated neurons in the MnPO was the highest in the sham group, followed by the local anesthesia and PRF groups, and the lowest in the CRF group. This order is the same as that for the central temperature at 90 min post-treatment. Taken together, the delayed recovery of body temperature in the nerve block groups suggested that this might be due to decreased neural activity of MnPO, a central thermoregulatory mechanism.

Long-term observations have provided insight into the long-term effects of nerve blocks on body temperature regulation. The transient temperature decrease observed exclusively in the CRF group at three hours post-treatment suggests a prolonged effect of CRF on thermoregulation, which may be due to more sustained nerve damage. However, the normalization of temperatures across all groups thereafter, and the lack of significant differences in c-Fos activity in the thermoregulatory centers at 48 h, suggest that the acute thermoregulatory disturbances caused by the nerve blocks are temporary and do not result in lasting alterations in central thermoregulatory functions. These findings underscore the resilience of the thermoregulatory system, and the transient nature of the thermal disturbances caused by peripheral nerve blocks.

This study has some limitations. An important limitation of this study is the relatively small sample size of three mice per group, which may not provide sufficient statistical power to detect smaller effect sizes. This was a deliberate choice made during the initial design phase of our experiment, aiming to balance the need for demonstrable results with ethical considerations regarding the use of mice. The main objective of this study, the effect of each treatment group on body temperature compared to the untreated group, was statistically significantly different. However, a larger sample size would be needed to examine significant differences between treatment groups. Moreover, since the procedure was performed under general anesthesia with isoflurane, making comparisons with local anesthesia-only conditions is challenging. General anesthesia itself affects body temperature; therefore, comparisons should also be made with the block method alone. Additionally, the degree of sensory and motor nerve damage caused by each nerve block was not evaluated. Furthermore, to analyze the differences in thermoregulation caused by different types of nerve blocks, the following factors must be considered, assuming similar block efficacy: type and amount of local anesthetic, voltage and duration of PRF and CRF, etc. Therefore, the differences in the effects between nerve blocks will be investigated in the future.

The effects of epidural and spinal administration of anesthesia on thermoregulation have been previously reported; however, the effects of peripheral nerve blocks have not been clarified^18^. Since hypothermia increases the risk of postoperative complications^19^, thermoregulation plays an important role in cases of prolonged surgery and treatment of severely ill patients ^20,21^. To minimize these risks and improve patient outcomes, a comprehensive understanding of the impact of peripheral nerve blocks on thermoregulation is warranted. This study revealed that different nerve block methods (local anesthesia, CRF, and PRF) have complex effects on central and peripheral thermoregulation. These results suggest that MnPO, the thermoregulatory center, may be involved in these phenomena. Although it remains to be seen how nerve block in the DRG affects the neural circuitry of thermoregulation to the MnPO via the spinal dorsal horn, our results offered valuable insights into the impact of peripheral nerve blocks on thermoregulatory mechanisms.

## Materials and methods

### Animal experiments

Eight-week-old C57B/6J male mice were purchased from the Japan Society for the Promotion of SLC (Shizuoka, Japan). A total of 12 mice were used in this study. The mice were housed under standard conditions, with temperatures maintained between 23 and 25 °C and subjected to a 12-hour day/night cycle, with libitum access to food and water. The mice were randomly assigned to one of four groups (6 mice per group): three nerve block groups (local anesthesia, CRF, and PRF groups), and a sham group. Temperature measurements were taken 10 min before treatment, immediately post-treatment, and at 30-, 60-, and 90-minutes post-treatment (Fig. 1a). We conducted two sets of experiments: one observing the acute phase up to 90 min, and another extending the observation to the chronic phase up to 48 h. The reason for setting the 90-minute mark is that by this point, the sham group had recovered to pre-procedure temperature levels, which was considered indicative of recovery from the temperature reduction due to general anesthesia and procedural stimuli. In the chronic phase of the observation, body temperature measurements were taken at 3-, 6-, 12-, 24-, and 48-hours. Rectal temperature measured using a mouse thermometer (KN-91-AD1687. Natsume Seisakusho Co., Ltd., Tokyo, Japan) under restraint (Fig. 1b) served as the central temperature, while the surface temperatures measured using a non-contact thermometer (TG8818N. Your Shop Co., Ltd. (Hyogo, Japan) at the left and right hind paw surfaces served as the peripheral temperatures. The brains were harvested after the 90-minute temperature monitoring period. The number of animals used was minimized, and tissues were collected following euthanasia under deep anesthesia induced using a combination of anesthetics (0.3 mg/kg medetomidine, 4.0 mg/kg midazolam, 5.0 mg/kg butorphanol) to reduce pain.

This study was approved by the Animal Experimentation Committee of the Osaka University School of Medicine (Approval No. 28-071-004) and in accordance with the National Institute of Health Guide for the Care and Use of Laboratory Animals. All animal experiments were conducted in accordance with the research protocol and ARRIVE guidelines. All efforts were made to minimize the number of mice and to decrease anguish. If abnormalities or hypothetical humane endpoints (for example, difficulty in feeding or watering, breathing problems, self-harm, rapid weight loss of ≥ 20% in a few days) were shown in the experimental mice, they were immediately euthanized by intraperitoneal injection of pentobarbital (200 mg/kg).

### Nerve block

Following incision of the mouse’s thigh under general anesthesia with 1.5% isoflurane, the muscle layer was dissected to expose the right sciatic nerve, a common site for assessing systemic effects of nerve block techniques due to its significant size and easily accessible location. Mice in the local anesthesia group were administered with 100 µL of 0.75% ropivacaine (Anapeine^®^, AstraZeneca, Japan; 2 mg/mL). Mice in the CRF and PRF groups were subjected to needle tip temperatures of 80 °C for 90 s or 42 °C for 6 min. The RF current was delivered in bursts of 20 milliseconds duration, followed by a silent phase of 480 milliseconds to allow for heat dissipation. The voltage was set to approximately 45 volts. The radiofrequency (RF) generator (Abbott Medical Japan, Tokyo, Japan) was used for both CRF and PRF procedures. The sham group was treated with a nerve blocking needle grounding only. All procedures were conducted in a room maintained at a consistent temperature of 23 °C to ensure stable environmental conditions and minimize any external influences on body temperature measurements. To ensure consistency in the effects of general anesthesia across all groups, 1.5% isoflurane was administered for 10 min.

### Immunostaining

The harvested brains were perfused with 4% paraformaldehyde in 0.1 M phosphate buffer (PB). Subsequently, they underwent further fixation in the same solution. The brains were then cryoprotected in 0.1 M PB containing 30% sucrose and then frozen on dry ice.

During the sectioning process, Nissl-stained sections were examined under a microscope, and the median preoptic nucleus (MnPO) was identified using the Mouse Brain in Stereotaxic Coordinates (2nd edition, Academic Press Elsevier, Amsterdam, Netherlands). The brain sections were treated with 0.3% Triton-X and 3% bovine serum albumin in 0.01 M phosphate buffered saline (PBS) to block nonspecific staining and increase permeability to antibodies. Subsequently, they were incubated with anti-c-Fos rabbit polyclonal antibodies (1:1000; catalog no. ab190289; Abcam Inc., Cambridge, MA) in the pretreatment solution at 4 ℃ overnight. After thorough washing in 0.01 M PBS, the sections were treated with anti-rabbit IgG antibodies (1:500; catalog no. A-21206; Thermo Fisher Scientific, Waltham, MA, USA) in 0.01 M PBS at 22 ± 2 °C for 1 h. After undergoing thorough washing in 0.01 M PBS, the sections were mounted on slides using PermaFluor (Thermo Fisher Scientific). The stained samples were then analyzed using a BX53 microscope (Olympus Corporation, Tokyo, Japan). The number of c-Fos positive cells was counted using three consecutive sections per a mouse.

### Statistical methods

In all studies, the Student’s t-test was utilized to compare the difference between the sham group and each of the other two groups. Results of the statistical analysis were considered significant when *p < 0.05, **p < 0.01, ***p < 0.001. Temperature measurements were compared to those of the sham group for the corresponding period. Results are expressed as mean ± standard deviation (SD).

## Data Availability

All relevant data are within the paper.
